# Modeling Study: Characterizing the Spatial Heterogeneity of the COVID-19 Pandemic through Shape Analysis of Epidemic Curves

**DOI:** 10.21203/rs.3.rs-223226/v1

**Published:** 2021-02-23

**Authors:** Anuj Srivastava, Gerardo Chowell

**Affiliations:** 1. Department of Statistics, Florida State University, Tallahassee, FL, USA.; 2. Department of Population Health Sciences, School of Public Health, Georgia State University, Atlanta, GA, USA.

**Keywords:** Covid-19 incidence rates, epidemic curves, statistical shape analysis, region clustering, pandemic trends, flattening curves

## Abstract

**Background:**

The COVID-19 incidence rates across different geographical regions (e.g., counties in a state, states in a nation, countries in a continent) follow different shapes and patterns. The overall summaries at coarser spatial scales, that are obtained by simply averaging individual curves (across regions), hide nuanced variability and blur the spatial heterogeneity at finer spatial scales. For instance, a decreasing incidence rate curve in one region is obscured by an increasing rate curve for another region, when the analysis relies on coarse averages of locally heterogeneous transmission dynamics.

**Objective:**

To highlight regional differences in COVID-19 incidence rates and to discover prominent patterns in shapes of incidence rate curves in multiple regions (USA and Europe).

**Methods:**

We employ statistical methods to analyze shapes of local COVID-19 incidence rate curves and statistically group them into distinct clusters, according to their shapes. Using this information, we derive the so-called *shape averages* of curves within these clusters, which represent the dominant incidence patterns of these clusters. We apply this methodology to the analysis of the daily incidence trajectory of the COVID-pandemic for two geographic areas: A state-level analysis within the USA and a country-level analysis within Europe during late-February to October 1^st^, 2020.

**Results:**

Our analyses reveal that pandemic curves often differ substantially across regions. However, there are only a handful of shapes that dominate transmission dynamics for all states in the USA and countries in Europe. This approach yields a broad classification of spatial areas into different characteristic epidemic trajectories. In particular, spatial areas within the same cluster have followed similar transmission and control dynamics.

**Conclusion:**

The shape-based analysis of pandemic curves presented here helps divide country or continental data into multiple regional clusters, each cluster containing areas with similar trend patterns. This clustering helps highlight differences in pandemic curves across regions and provides summaries that better reflect dynamical patterns within the clusters. This approach adds to the methodological toolkit for public health practitioners to facilitate decision making at different spatial scales.

## Background

The ongoing pandemic of novel coronavirus disease (COVID-19) that erupted in China in December 2019 continues to generate substantial morbidity and mortality impact around the world [5,26,27]. As the novel coronavirus continues its march around the world, the daily trajectory of the epidemic in terms of new cases or deaths represents a key tool for epidemiologist and public health scientists to quantify the reproduction number, assess the evolution of the doubling time, and evaluate the impact of social distancing strategies in different parts of the word [27, 4]. The temporal evolution of daily counts of reported cases forms longitudinal data or *pandemic curves* that are crucial in evaluating the spread and growth dynamics of the pandemic [2,7,8,9,10,12,14,16,17,24]. However, the commonly used summary curves, obtained by accumulating or averaging pandemic curves collected over large regions, can hide substantial differences in transmission dynamics that exist at finer spatial scales [8]. These nuanced patterns associated with individual regions may be critical to inform the type and intensity of interventions to bring the epidemics under control [19,24].

This motivates the need to better understand and characterize spatial differences in the trajectories of the COVID-19 pandemic in different geographic areas around the world [26,27]. Indeed, the epidemic curves across different continents or countries may display completely different dynamics at a given time [4] and this motivates the need for spatial epidemiology methods [4,11]. At broader spatial sales, such dynamics include increasing trends, a leveling or stationary incidence pattern, and decreasing trends. At a finer level, the growth may be characterized by multiple modes depicting multiple waves of the epidemic [4]. Similarly, the epidemic curves at the subnational level within a country may also display different dynamics over time. The levels of pandemic can drastically change over time, depending upon the growth and decline cycles of the disease at a location. Because the type and intensity of public health interventions are expected to vary across space, characterizing the spatial-temporal dynamics is an essential first step [3,19,25]. Next, classifying and summarizing the spatial-temporal dynamics of the novel coronavirus in local areas is key for real-time public health decision making. The resulting epidemic features can then be linked to several other covariates, such as hospital capacity, testing facilities, equipment quality, personnel training, contact tracing, to name a few, that influence the course of the pandemic at local scales [1]. In this paper we focus only on the features of the epidemic curves and how we can use them to characterize spatial heterogeneity across spatial units. Epidemic curves provide important information about the trajectory of the spread albeit they hide substantial information about the spatial-temporal distribution of the cases in the population of interest.

While researchers track several variables for quantofying COVID-19 dynamics, we focus in this paper on the daily counts of new positive tests as daily functions of time. The same analysis can also be performed with other variables, such as the death counts, hospitalizations, and recovered patients also. The temporal changes in daily new positive tests, for different regions and communities, forms functional data or *pandemic curves*. Quantitative methods for analyzing functional data, termed *Functional Data Analysis* (see e.g., [18]), can help compare and investigate the diversity of the dynamics of transmission of the COVID-19. They can provide an objective framework to characterize the spatial-temporal dynamics of the epidemic in different geographic areas within the same country. In the past, functional data analysis has been used to study biological structures [13], stock markets trends, weather patterns, medical diagnostics [28], growth rates [20], and speech data. In the context of the COVID-19 pandemic, clustering of curves has been used to analyze travel patterns of migrants in China [6].

### Motivation for Shape Analysis

Overall trends in a pandemic are often obtained by summarizing pandemic curves. Data scientists tend to prepare summaries by simply averaging data across experimental units. Similarly, national or continental reports for COVID-19 developments are typically prepared by averaging or aggregating curves (whether cumulative counts, or new positives, or deaths) over different counties, states, and countries. This has the potential for obscuring important trends present in the individual curves characterizing transmission at finer spatial scales. For instance, a decrease in infections in one region can be negated by an increase in another; together their average will appear as roughly constant over a period of time. To handle this issue this paper advocates the averaging of “shapes” of curves, rather than the raw curves themselves. This leads to more natural solutions for aggregating and averaging curves.

While epidemic curves have been analyzed statistically in the past, the focus on their shapes is a relatively new concept. Past research has mainly focused on estimation and prediction of the curves [2,7,9,14,16,17] rather than on their analysis.

**Figure 1:**
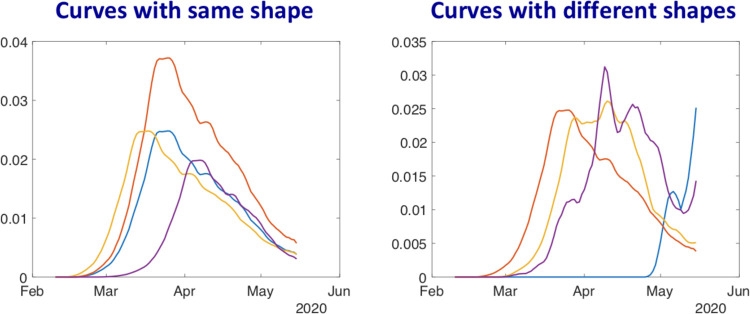
Demonstrating the concept of shapes of curves: All the curves in the left panel are deemed to have the same shape, as they differ only in their vertical scales and horizontal shifts. The curves in the right panel have different shapes.

We motivate this new focus by answering the question: *Why is it interesting to study shapes of these curves*? By focusing on shapes, one is more interested in the numbers and relative heights of peaks and valleys in a curve, rather than their precise locations. For instance, all bell-shaped curves will be deemed similar in shape even if their peaks are located at slightly shifted points and the peak heights are different. These bell curves will be considered different from other shapes, say monotonically increasing curves. We point out that a comprehensive handling of the diversity of shapes (of pandemic curves) makes this paper different from some past works, for example [9], where all epidemic curves are modeled as bell-shaped functions and are classified into different classes according to their means and dispersion. The curves with different up and down patterns will also be considered different irrespective of the locations of their crossover points. (Figure 1) illustrates this idea pictorially. In the left panel we see a number of curves that differ only in heights and horizontal shifts. All these curves are deemed to have the exact same shape despite differences in the corresponding y-values. In the right panel we see curves with different numbers, locations, and relative heights of the modes. These curves are deemed to have different shapes and one can quantify the shape differences using tools from shape analysis.

**Figure 2:**
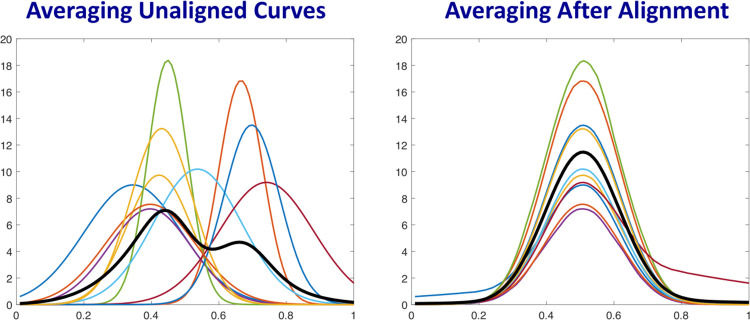
Averaging curves: The left panels shows ten curves in different colors and their classical Euclidean average in black. The right panel shows these curves after temporal alignment and the new average in black. This new average is called their shape average.

A further motivation for analyzing shapes of curves is presented through an example in (Figure 2). The left panel of this figure shows ten smooth unimodal curves in different colors and their classical Euclidean average in black. Suppose that these colored curves depict incidence rates for ten different states, over a certain observation time period. Even though each of these individual curves have a similar, well-defined peak, their average shows a bimodal shape. Thus, this average curve does not provide a good summary or representation of the original data. This discrepancy is due to the fact that the locations of peaks in different curves are different and this misalignment blurs out the peaks in the averaging. The solution is to use a (nonlinear) alignment algorithm that time warps these functions and aligns their peaks. Time warping is a technique to modify the time domain of a curve that results in moving points on that curve horizontally, without points crossing each other. Some part of the time interval gets stretched while other gets shrunk, keeping the overall interval size the same. Once the peaks are aligned across curves using time warping, we can compute their average (and other statistics), as illustrated in the right panel. This is called an *averaging of the shapes* of curves.

By comparing the *shapes* of epidemic curves, each representing the transmission dynamics in different geographic areas within the same country or the same continent, we can classify these curves into groups or clusters that exhibit a similar growth-decline pattern. This approach offers multiple benefits. Indeed, it allows us to compute overall averages that better reflect the actual growth patterns of the states. Secondly, and more importantly, it helps label each state in terms of the state of the pandemic. It can also help us discover predominant patterns in epidemic growth, using data across different locations, times, and scales. These shapes can, in turn, be used in further statistical and modeling analyses, e.g. evaluating the effects of countermeasures.

The next question is: *How does one quantify and statistically analyze shapes of rate curves*? Mathematically, shape is a property that remains unchanged if we rescale axes or translate the curves along vertical or horizonal axes. In fact, one even allows nonlinear time warping of the time axis, resulting in uneven horizontal shifts of the peaks and valleys, to be deemed shape-preserving transformations. The invariance of shape to such transformations makes shape analysis a difficult problem. In order to compare and analyze shapes of multiple curves, one has to standardize their domains by scaling axes, normalizing heights, and aligning their peaks and valleys using time warping functions (as shown in the right panel of Figure 2). The resulting curves can then be analyzed for shapes. We employ a well-established methodology for shape analysis of functional data, introduced and described in [20]. This framework, called *elastic functional data analysis*, provides comprehensive tools for generating statistical summaries and modeling of curves while focusing only on their shapes. We apply these tools to incidence rate curves of the COVID-19 pandemic with the goal of providing a well-defined framework to guide public health decision making at different spatial scales. The following sections describe the methodology to analyze and cluster rate curves of COVID-19 reported cases. We first pre-process the data into smooth incidence rate curves for each local unit (a state or a country) over the observation interval. Then, we analyze shapes of these rate curves to compare, cluster and summarize growth rates.

### Specific Aims

In this paper we seek to:

1. Adapt techniques for shape analysis of curves to compare and cluster pandemic curves associated with different regional units (counties in a state, states in a county, or countries in a continent).
2. Generate representative epidemic curves at cluster level, thus avoiding loss of information that results from aggregating local epidemic curves at coarser spatial scales.
3. Apply this methodology to the analysis of the daily incidence trajectory of the COVID-pandemic at two spatial scales: A state-level analysis within the USA and a country-level analysis within Europe during mid-February to mid-October 2020.
4. Provide regional summaries that may help guide public health decision making at different spatial scales.

## Methods

Next we describe the methodology used to analyze and cluster COVID-19 incidence curves. The method involves pre-processing raw count data to produce smoothened and normalized incidence curves and methods for analyzing their shapes. We start by providing a step-by-step listing of the overall procedure.

### Overview of the Procedure

We start by providing a step-by-step overview of the process of discovering dominant patterns of pandemic growth in different regions. The analysis consists of the following steps:

1. **Pre-Processing Step**: Start with (daily update of) the cumulative count data of positive COVID-19 tests for each region and perform pre-processing to result in normalized and smoothed incidence rate curves.
2. **Temporal Alignment Step**: Perform alignment of all curves in a country or continent using nonlinear time warping, in order to align their peaks and valleys.
3. **Distance Computation Step**: Compute pairwise distance between aligned curves to result in a full distance matrix.
4. **Clustering Step**: Perform clustering of regions into a small number of subgroups or cluster using this distance matrix.
5. **Mean Shape Computations**: Re-align curves within each cluster and compute the average of the aligned curves. This average shape is used as a representative or a summary of incidence rate curves in that cluster. We also compute one standard-deviation bands around the mean curves as a measure of confidence.

This process classified all spatial units into a pre-determined number of clusters and their corresponding average incidence-rate curves.

### Pre-Processing Step

**Figure 3:**
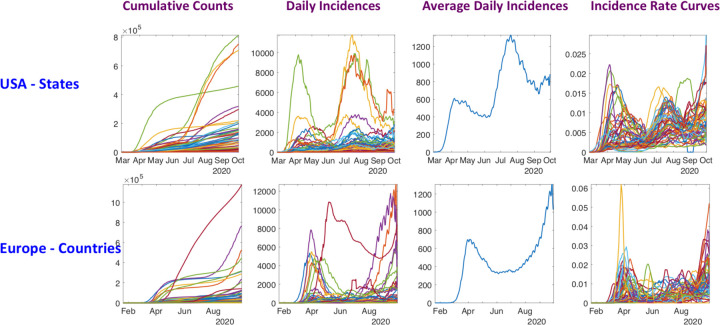
Preprocessing COVID-19 data into growth rate functions. From left to right: Original positive test data; Curves of daily new cases; Smoothed and scaled rate curves; Average of rate curves. The top row shows data for 51 American regions (50 states + DC) and bottom row shows the data for 53 European countries (after removing some outliers and missing data).

The following three steps constitute pre-processing applied to raw COVID-19 daily count data for each region individually. We start with the cumulative counts of people testing positive on a daily basis. This data for USA and Europe is shown in the leftmost column of (Figure 3).

- **Time-Differencing**: Since the data includes cumulative counts (or total number) of positive test counts for different regions, we first calculate time differences (approximating time derivatives) of the data to reach daily new incidence counts. If 𝑓_i_(𝑡) denotes the given cumulative positive counts for state *i* at time *t*, then the per-day incidence-rate for that state at time *t* is given by 𝑔_i_(𝑡) = 𝑓_i_(𝑡) − 𝑓_i_(𝑡 − 1). These rate curves for US States and European countries are shown in the second column of (Figure 3). The third column shows the classical Euclidean average of these curves, as a representative of the pandemic situation for the whole country or the whole continent.
- **Re-Scaling**: The volumes of incidence rates for different regions are very different, depending upon population counts, densities, and other variables. In order to focus on the shapes of the curves, we rescale each curve as follows. We compute the total positive tests for a state over the observed time interval, *i.e. $r_{i}=\sum_{t}g_{i}\left(t\right)$* and then we define the re-scaled curves to be $h_{i}\left(t\right)=g_{i}\left(t\right)/r_{i}$.
- **Smoothing**: Next, we smooth these normalized growth rate curves using the smooth function in Matlab. This smoothing is performed to reduce observation noise and to help focus on the overall shape of the curve. With a slight abuse of notation, we shall call the resulting functions ℎ_i_(𝑡) also. These are the smoothed and normalized growth rate curves, or simply rate curves henceforth. The last column of (Figure 3) shows these rate curves for the two populations: USA states (top) and European countries (bottom).

### Temporal Alignment and Clustering of Incidence Curves

The resulting smoothed and normalized rate curves are then used in statistical analyses. There are several possibilities for this analysis, including modeling, testing, prediction [7, 12,16] and classification [17]. As mentioned earlier, a raw averaging of data across all states is bound to smooth over interesting patterns and lose interesting smaller structures. The third column of (Figure 3) show averaging of the daily counts of regions for each dataset (USA and Europe). Looking at these average curves, one gets the impression that the incidence rate is either declining or rising universally at any time. However, this conclusion overlooks the local heterogeneity of dynamics in different regions. Consequently, it is difficult to justify the use of overall averages as representatives of pandemic patterns.

Since our main goal is to analyze shapes of rate curves for different regions, we start by temporally aligning these curves. This process of alignment of curves is also called *phase-amplitude separation* [15]. There are several algorithms present in the literature for alignment of functions. In this paper we use the elastic alignment algorithm introduced in [21] and described in Chapter 8 of Srivastava and Klassen [20]. The basic idea behind this approach is as follows. Let ℎ_i_, ℎ_j_ represent two rate curves that need to be aligned temporally. In other words, time warp curve(s) in such a way that their peaks and valleys are co-located. The time warping function is given by γ, a smooth, monotonically increasing function on the observation interval. For a pandemic curve ℎ_j_, the composition ℎ_j_ ∘ γ is called the time warping of ℎ_j_. For alignment purposes, one defines a transformation of a curve, called the square root velocity function (SRVF), as follows: for any curve ℎ: [0,1] → 𝑅, define its SRVF using $q\left(t\right)=sign\left(\dot{h}\left(t\right)\right)\sqrt{\left|\dot{h}\left(t\right)\right|}$, where $\dot{h}\left(t\right)$ denotes the time derivative of ℎ. To align ℎ_j_ with ℎ_i_, one solves the optimization problem: $\underset{\gamma }{min}\left\|q_{i}-\left(q_{j}\circ \gamma \right)\sqrt{\dot{\gamma }}\right\|$, where ‖.‖, denotes the L^2^ norm of a function. That is, $\left\|q\right\|=\sqrt{\left(\sum_{t}q{\left(t\right)}^{2}\right)}$. The minimization is performed using the well-known Dynamic Programming algorithm as described in [19]. The optimal warping γ* is then applied to ℎ_j_, according to ℎ_j_ ∘ γ*, to obtain the desired alignment. To align multiple rate curves one applies this idea repeatedly, by applying each given curve to an overall mean.

A simulated illustration of this alignment process is shown in (Figure 2). The left panel of the figure shows ten unaligned curves and the right panel shows the same curves aligned using time warping as mentioned above. One can see that the peaks and the slopes of these curves are very well aligned. We perform a similar alignment of COVID-19 incidence curves, with results shown later.

Next we cluster these aligned curves into smaller, homogeneous groups. This clustering is important in that it helps recognize spatial heterogeneity of growth rates across geographical regions. For the purpose of clustering, we use a simple metric to compare any two curves. For any two *aligned* rate curves, ℎ_i_ and ℎ_j_, we simply compute the norm $\left\|h_{i}-h_{j}\circ \gamma *\right\|$, where the bars denote the L^2^ norm of the difference function and γ ∗ is the time warping that aligns ℎ_*j*_ to ℎ_*i*_. To perform clustering of rate curves into smaller groups, we apply the dendrogram function in Matlab using the “ward” linkage. The number of clusters is decided empirically based on the display of overall clustering results. We elaborate on this further later on in the results section.

### Averaging of Growth Curves within Clusters

Once we have clustered regions into different clusters, we seek to derive an appropriate average or a representative curve for each cluster. As earlier, a simple arithmetic averaging of curves is not always the best option. Instead, we use the alignment process *once again* to align curves within each cluster. Once the curves are aligned, we compute averages of these aligned curves to reach the cluster average. These cluster averages are better indicators of the trends in pandemic evolution, as compared to the overall averages that are often shown in current statistical summaries.

### Publicly Available Code

The general procedure for shape analysis of curves is freely available in an R code package fda srvf [23]. The specific tools presented in this paper have been developed using Matlab and can be found in the github repository *EpiCurvesShapeAnalysis*.

### Data

We analyze daily series of reported COVID-19 cases at two different levels of spatial aggregation: States within the USA and countries in Europe.

For the USA analysis, we retrieved daily cumulative case count data from the COVID Tracking Project, a volunteer organization dedicated to collecting and publishing data on the spread of COVID-19 in the United States [22]. Data from multiple sources, such as state or district health departments, and trusted news reports, are compiled and assessed for data quality to report the best available data for each state. Here we use reported daily state- and national-level cumulative case counts from February 27^th^, 2020 to October 1^st^, 2020.

For the country-level analysis in Europe, we retrieved the data from World Health Organization: Coronavirus disease (COVID-2019) situation reports on October 1^st^, 2020 [26].

## Results

The following sections describe the results of our analyses using state-level data for the USA and country-level data for Europe.

### State Level Analysis for the USA

**Figure 4:**
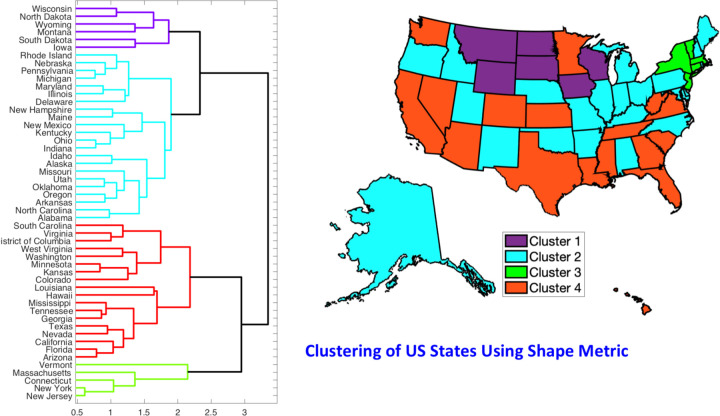
Clustering of US states according to their normalized growth rate curves for the COVID-19 pan demic. The left side of the figure shows a dendrogram plot -- a hierarchical clustering of states – obtained using the Dendrogram function in Matlab. The resulting four groups are shown in different colors in the d endrogram plot. The right side of the figure shows a color coding of these states according to their clusters. States drawn in the same color belong to the same cluster.

The normalized incidence rate curves for US States, for the time period 2/27/2020 to 10/1/2020, are shown in the top-right panel of (Figure 3). The results for clustering these 51 curves are shown in (Figure 4). It shows that there are four predominant clusters, which we consider for further analyses. We could also choose five clusters instead, but the results do not change significantly. The listing of states according to this clustering is as follows.

- **Cluster 1**: Iowa, Montana, North Dakota, South Dakota, Wisconsin, Wyoming.
- **Cluster 2**: Alabama, Alaska, Arkansas, Delaware, Idaho, Illinois, Indiana, Kentucky, M aine, Maryland, Michigan, Missouri, Nebraska, New Hampshire, New Mexico, North C arolina, Ohio, Oklahoma, Oregon, Pennsylvania, Rhode Island, Utah.
- **Cluster 3**: Connecticut, Massachusetts, New Jersey, New York, Vermont.
- **Cluster 4**: Arizona, California, Colorado, District of Columbia, Florida, Georgia, Hawa ii, Kansas, Louisiana, Minnesota, Mississippi, Nevada, South Carolina, Tennessee, Tex as, Virginia, Washington, West Virginia.

**Figure 5:**
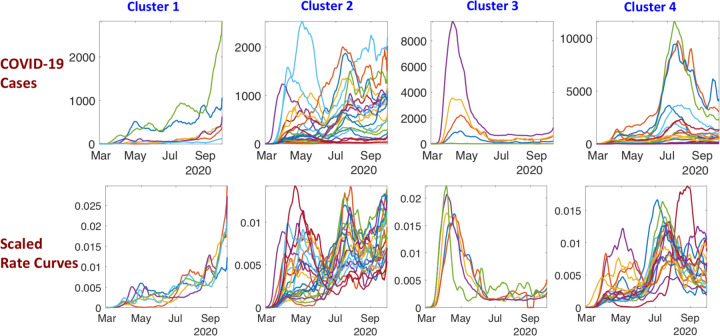
Incidence rate curves for 51 US states clustered in four groups. Top row shows the incidence rates (daily positives) at their original scales while the bottom row shows the normalized curves.

The incidence rate curves for these clustered states are shown in (Figure 5), both before (top) and after (bottom) normalization. Since curves for different states have quite different scales, it is not easy to discern overall shape patterns, even within the same cluster in the top row. After rescaling the curves to the same scale, the general trends in the growth rates become clearer.

**Figure 6:**
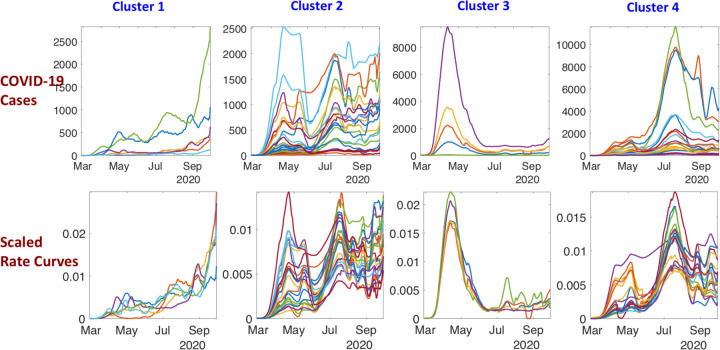
Aligned incidence rate curves for 51 US states clustered in four groups. Top row shows the curves at their original scales while the bottom row shows the normalized curves. For example, for states in Cluster 3, the rate fist goes up sharply, then comes down sharply and stays low. For states in Cluster 1, the rate is low at first but starts increasing rapidly as time progresses; these are the states with most concern at this time stage. States in Cluster 2 are characterized by multiple high-level peaks and the trend continues to show high incidence rates. For states in Cluster 3, the first peak is relatively small, but the second peak is quite high. The trends here suggest a smaller third peak towards the end of the observation period.

**Figure 7:**
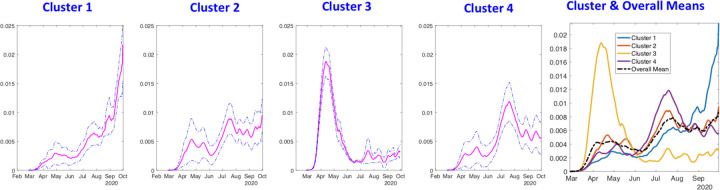
Average shapes of the growth rate curves, along with a one standard-deviation band around the mean, in each of the four clusters for the state-level USA analysis. The last panel shows all cluster averages together with the overall mean. For Cluster 1: the rate is low at first and then climbs rapidly; for Cluster 2, there are two waves with similar intensity; for Cluster 3, the rate climbs rapidly and then comes down rapidly; and, for Cluster 4 the rate shows two waves with the second wave being much larger and dominant. The rightmost panel of this figure shows all the cluster averages in the same plot, to help visualize their differences.

Although the shapes of curves within a cluster are quite similar, the averaging of these curves still can lose structure. As mentioned earlier, one needs to align the peaks and valleys of curves before averaging. To further extract typical trends, we use re-align rate curves within each cluster. (Figure 6) shows these aligned rate curves in each cluster. It is much easier to infer pandemic trends in the aligned and scaled data.

(Figure 7) shows the average rate curves for each cluster. We obtain these curves by aligning and averaging rate curves in each cluster separately. It can be seen clearly that the pandemic growth patterns are very distinct in the four clusters. Furthermore, the overall mean shown in the rightmost plot seems to have lost a lot of structure due to gross aggregation, when compared to these cluster means. This overall mean corresponds to the methods currently used to summarize epidemic curves using simple arithmetic averaging.

### Country-Level Analysis in Europe

**Figure 8:**
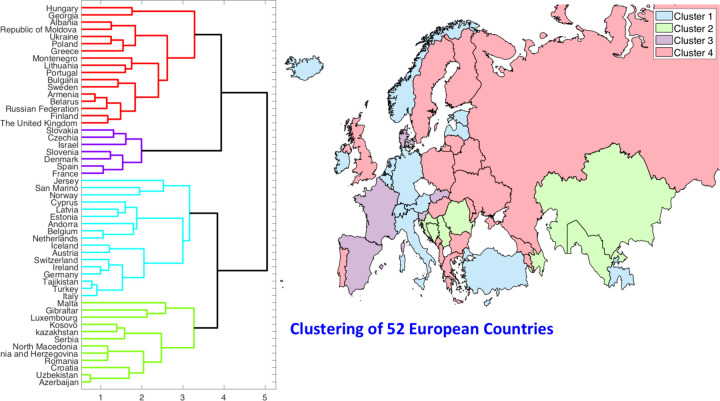
Clustering of European countries according to their normalized growth rate curves. The left side shows their clustering using a dendrogram while the right side of the figure shows a color coding of the countries according to their cluster memberships.

Now we present clustering and shape analysis of rate curves for 53 European countries. The pre-processed incidence rate curves were shown previously in the bottom row of (Figure 3). In this case we omit some intermediate results from clustering and alignment steps, and directly present the final clustering results. A dendrogram-based hierarchical clustering of countries is presented on the left side of (Figure 8). We choose to divide these countries into four clusters, as shown in the figure.

The cluster membership of 53 countries is listed below:

- **Cluster 1**: Italy, Germany, Switzerland, Norway, Austria, Netherlands, Iceland, Andorra, Belgium, Estonia. Ireland. Latvia, San Marino, Cyprus, Turkey, Jersey, Tajikistan.
- **Cluster 2**: Croatia, Azerbaijan, Romania, Bosnia and Herzegovina, Luxembourg, North Macedonia, Serbia, Gibraltar, Malta, Kazakhstan, Uzbekistan, Kosovo.
- **Cluster 3**: France, Spain, Israel, Czechia, Denmark, Slovenia, Slovakia.
- **Cluster 4**: United Kingdom, Sweden, Georgia, Greece, Finland, Russian Federation, Portugal, Belarus, Hungary, Armenia, Lithuania, Poland, Ukraine, Bulgaria, Republic of Moldova, Albania, Montenegro.

**Figure 9:**
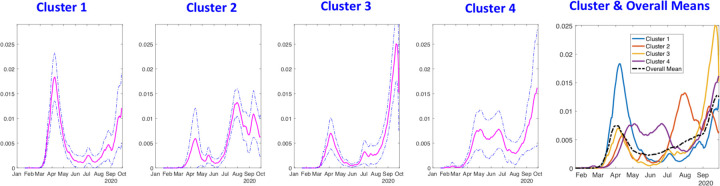
Average shapes of the growth rate curves in each of the four clusters in Europe. The last panel shows all averages together with the overall mean. For cluster 1, the rate climbs rapidly, comes down all the way to the normal levels and then starts showing the second wave. For cluster 2, the rate shows multiple waves and the rates coming down after the second wave. In cluster 3, the one with the most concern, the second wave shows an exponential rise in the incidence rates. In case of cluster 4, the incidence rate is still climbing rapidly during the second wave. The rightmost panel shows all the cluster averages in the same plot.

Once the curves are aligned within their clusters, it is easier to visualize the common peaks and valleys (highs and lows) in each group. (Figure 9) shows the average normalized growth curves for each cluster. We obtain these curves by aligning and averaging normalized curves in each cluster individually. The growth patterns are very distinct in the four clusters. Similar to the USA results, the individual cluster means are quite different from the overall mean shown in the rightmost panel. Once again, this demonstrates the superiority of using shape-based clustering and cluster means as representations of epidemic curves, rather than using overall means.

## Discussion

This paper develops an approach for clustering and analyzing shapes of incidence rate curves of the COVID-19 pandemic, with a focus on highlighting the spatial heterogeneity that exists at finer spatial scales. Specifically, we have applied this methodology to characterize the dynamics of the pandemic at two different scales of spatial aggregation: Across states within the USA and across nations within Europe. The main findings of this paper are as follows. The shapes of incidence rate curves are different across the spatial units, but they often cluster into a small number of dominant groups with characteristic patterns. The resulting broad classifications of the shapes (e.g., rapidly rising and then rapidly lowering, or low at first and then rapidly rising, or multiple dominant peaks, or multiple peaks with different intensities) are clearly visible and very meaningful. Nearby states, e.g. New York, Connecticut, and Massachusetts, are often found to follow similar dynamics. Computing average growth rates within the clusters is more appropriate than taking raw averages across all spatial areas comprising the population, particularly when the epidemic is comprised of asynchronous outbreaks.

Our state-level analysis indicate that during this period Connecticut, Massachusetts, New Jersey, New York, and Vermont are the only states that appear to be bringing the COVID-19 epidemic under control at this stage (Cluster 3). The other three characteristic patterns that emerge from our analysis paint a grim picture of the COVID-19 epidemic in the USA at a time when most of the states have reopened their economies at least in some way. Similarly, our country-level analysis of the epidemic in Europe reveals that pandemic rates have started rising rapidly and very few countries appear to have the epidemic under control at this stage. While many of these countries had low rates during the later summer months, the counts are growing in almost all countries. Countries in Cluster 2 are characterized by a small downward trend at the moment and appear to be in process of controlling their epidemics. In contrast, the epidemic is following an alarming increasing trend in France, Spain, Israel, Czechia, Denmark, Slovenia, and Slovakia. Several other countries (Cluster 3 and Cluster 4) show a slow but steady increase in the incidence rates.

These results are for the period 02/27/20 – 10/01/20 and, of course, the results can change as the data evolves over time. While these results are applicable to the time period ending at 10/01/20, the results will change as new data arrives. The changes in clustering and cluster membership of states and countries, as data changes over time, can be an interesting future study under this framework.

An important use of this discovery of dominant patterns is in prediction of future courses of pandemics for different regions. For instance, one can try to predict the *shape* of future pandemic curve for a state, by predicting which cluster is it going to belong too. Since there are only a handful of dominant patterns, this task becomes easier with our analysis.

It is worth noting that the methodology employed here requires little human intervention. The only quantity that may be specified manually is the number of clusters, and that is left as a choice for the end user to explore in sensitivity analyses. Hence, the clusters of spatial units that emerge from the analysis provides an objective classification system of epidemic patterns, which can be used to guide the implementation or relaxation of public health measures as the epidemic emergency evolves over time.

While we have focused our analysis on the time series of confirmed cases across spatial areas, our analysis could be extended to consider the trajectory of the epidemic in terms of the number of reported COVID-19 deaths or hospitalizations.

### Limitations of the Method

One limitation of our analysis stems from the fact that testing rates have generally improved across spatial areas during the course of the pandemic, which could have influenced the shapes of the epidemic curves especially during the early transmission phase. In particular, the process of ramping up testing rates took several weeks in the USA, and accumulating evidence suggests that the epidemic in the USA and Europe likely started much earlier than initially thought. On the methodological side, our framework assumes that the full data is available to ascertain shapes of incidence curves. For regions, where the pandemic patterns are not yet fully evolved and shapes are not yet fully formed or observed, it is difficult to compare with states that are more advanced in the pandemic. This is a major limitation in the sense that we assume that all the regions must be similarly situated in terms of pandemic progression.

## Conclusion

In conclusion, we have employed a statistical framework to identify and classify spatial heterogeneity of the COVID-19 pandemic based on incidence rate curves at different spatial-temporal scales. By focusing on the shapes of incidence curves, this framework allows us to cluster different regions (e.g. states in a country or countries in a continent) into smaller groups of regions that follow similar patterns and provides a basis for the implementation of spatially relevant public health interventions. Thus, the output could be readily used by policy makers and public health officials to identify geographic regions of particular concern and facilitate the allocation of scarce resources. For instance, one can focus resources on clusters that show a rising trends in the incidence rates, relative to the areas that are trending downwards in incidence rates. Since the pandemic data and consequently shapes of pandemic curves change over time, this calls for regular updates in clustering and classifications of different states and countries. Moreover, the changes in cluster memberships over time could guide health officials about long range trends and phenomena.

Another potential use of the average shapes obtained for different clusters is to help predict the future course of the pandemic, but in a region-by-region manner. The past patterns of pandemic waves may be useful in predicting future trends when combined with statistical prediction methods.
